# Smoking-associated lung cancer prevention by blockade of the beta-adrenergic receptor-mediated insulin-like growth factor receptor activation

**DOI:** 10.18632/oncotarget.12342

**Published:** 2016-09-29

**Authors:** Hye-Young Min, Hye-Jin Boo, Ho Jin Lee, Hyun-Ji Jang, Hye Jeong Yun, Su Jung Hwang, John Kendal Smith, Hyo-Jong Lee, Ho-Young Lee

**Affiliations:** ^1^ Creative Research Initiative Center for Concurrent Control of Emphysema and Lung Cancer, College of Pharmacy, Seoul National University, Seoul 08826, Republic of Korea; ^2^ Department of Molecular Medicine and Biopharmaceutical Science, Graduate School of Convergence Science and Technology, Seoul National University, Suwon 16229, Republic of Korea; ^3^ College of Pharmacy, Inje University, Gimhae 50834, Republic of Korea; ^4^ Department of Thoracic Head & Neck Medical Oncology, The University of Texas M. D. Anderson Cancer Center, Houston, Texas 77030, USA; ^5^ College of Pharmacy and Research Institute of Pharmaceutical Sciences, Seoul National University, Seoul 08826, Republic of Korea; ^6^ Interdisciplinary Program in Genetic Engineering, Seoul National University, Seoul 08826, Republic of Korea

**Keywords:** 4-(methylnitrosamino)-1-(3-pyridyl)-1-butanone, insulin-like growth factor 1 receptor, insulin-like growth factor 2, β-adrenergic receptor, lung cancer

## Abstract

Activation of receptor tyrosine kinases (RTKs) is associated with carcinogenesis, but its contribution to smoking-associated lung carcinogenesis is poorly understood. Here we show that a tobacco-specific carcinogen 4-(methylnitrosamino)-1-(3-pyridyl)-1-butanone (NNK)-induced insulin-like growth factor 1 receptor (IGF-1R) activation via β-adrenergic receptor (β-AR) is crucial for smoking-associated lung carcinogenesis. Treatment with NNK stimulated the IGF-1R signaling pathway in a time- and dose-dependent manner, which was suppressed by pharmacological or genomic blockade of β-AR and the downstream signaling including a Gβγ subunit of β-AR and phospholipase C (PLC). Consistently, β-AR agonists led to increased IGF-1R phosphorylation. The increase in *IGF2* transcription via β-AR, signal transducer and activator of transcription 3 (STAT3), and nuclear factor-kappa B (NF-κB) was associated with NNK-induced IGF-1R activation. Finally, treatment with β-AR antagonists suppressed the acquisition of transformed phenotypes in lung epithelial cells and lung tumor formation in mice. These results suggest that blocking β-AR-mediated IGF-1R activation can be an effective strategy for lung cancer prevention in smokers.

## INTRODUCTION

Lung cancer is the main cause of cancer-related human death in Korea and worldwide [1–3]. The development of molecular targeted anticancer agents has resulted in better therapeutic responses in some types of lung cancer, including non-small cell lung cancer (NSCLC) with mutant epidermal growth factor receptor (EGFR) [4]. Nevertheless, drug resistance reduces the effectiveness of these targeted anticancer drugs [5], and the 5-year survival of lung cancer remains less than 20% [2]. In this regard, cancer chemoprevention, an approach to prevent, retard or reverse the carcinogenic process using dietary or synthetic chemicals [6, 7], can be an effective strategy for controlling lung cancer. Lung carcinogenesis is a multi-step, multi-path, and multi-focal process [6]. Hence, chemopreventive agents are anticipated to control each step of carcinogenesis to prevent normal cells from carcinogen-mediated genetic or epigenetic alterations and to suppress clonal expansion, proliferation, invasion, and metastasis of malignant cells. Numerous clinical trials investigating the effectiveness of lung cancer chemoprevention have been conducted using several natural or synthetic chemicals, including aspirin, β-carotene, vitamin E, and selenium [7]. However, the chemoprevention trials for lung cancer to date have shown negative or somewhat harmful effects; currently, no agents have been identified to be effective for the prevention of lung cancer. These findings suggest the necessity to discover novel strategies to control lung cancer.

Tobacco smoking (TS) is responsible for the majority of NSCLC. Therefore, understanding the signaling changes involved in tobacco carcinogen-mediated lung carcinogenesis is important to identify appropriate targets for lung cancer chemoprevention. Epidermal growth factor receptor (EGFR) is a plausible target [8]; however, *EGFR* mutations are primarily found in lung cancer in never-smokers [9]. *KRAS* is frequently mutated in smoking-related lung cancer [9]; however, the failure of the development of anti-Ras inhibitors [10] has dampened the enthusiasm for targeting *KRAS*. Hence, the development of novel targets involved in TS-mediated lung carcinogenesis is an appropriate strategy for lung cancer chemoprevention.

In the present study, we investigated the mechanisms of tobacco-specific nitrosamine 4-(methylnitrosamino)-1-(3-pyridyl)-1-butanone (NNK)-induced lung tumor formation. We found that NNK triggered insulin-like growth factor 1 receptor (IGF-1R) phosphorylation via β-adrenergic receptor (β-AR)-mediated increases in *IGF2* transcription. Treatment with β-AR antagonists suppressed NNK-induced IGF-1R phosphorylation, significantly inhibiting NNK-stimulated lung epithelial cell transformation and murine lung tumorigenesis. These results suggest that suppression of IGF-1R activation by blockade of β-AR can be an effective approach to prevent smoking-induced lung cancer.

## RESULTS

### NNK increased IGF-1R phosphorylation in human lung epithelial cells

**Figure 1:**
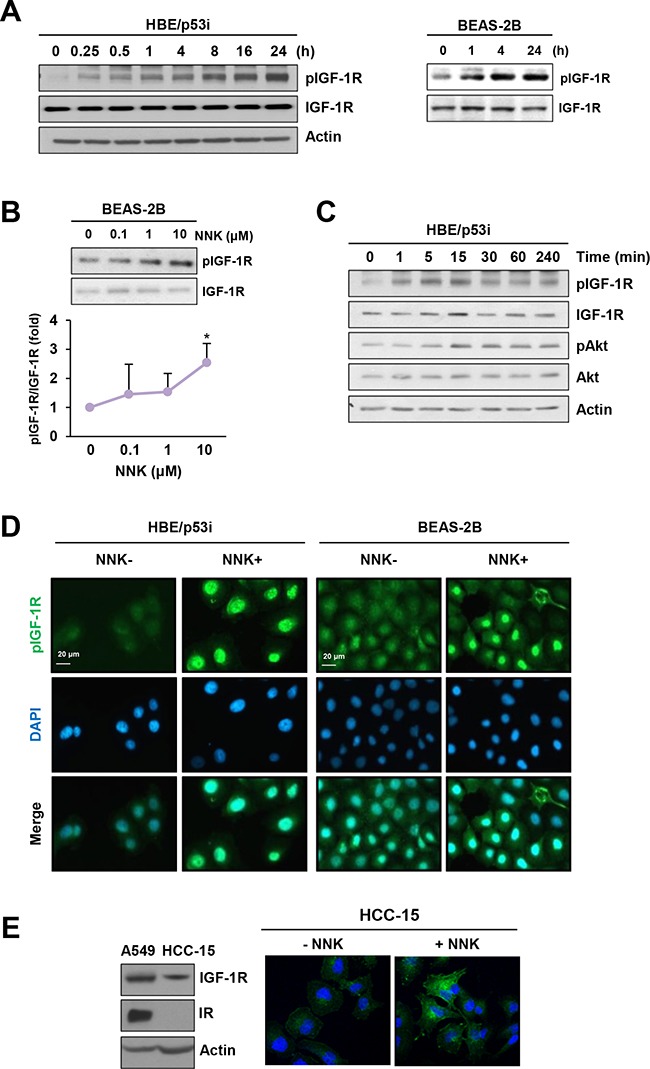
NNK induces IGF-1R phosphorylation **A.** Immunoblot analysis demonstrating a time-dependent increase in IGF-1R phosphorylation by treatment with NNK (10 μM) in HBE/p53i and BEAS-2B cells. **B.** Immunoblot analysis demonstrating a dose-dependent increase in IGF-1R phosphorylation by treatment of BEAS-2B cells with NNK (10 μM) for 24 h (*n* = 3 per group). Densitometric analysis of total and phosphorylated IGF-1R blots (normalized to actin) was performed using the Image J software. Data are presented as the mean ± SD. The statistical significance of difference was determined by Student's *t*-test (*: *P* < 0.05). **C.** Immunoblot analysis demonstrating a time-dependent increase in the phosphorylation of IGF-1R and Akt by treatment with NNK (10 μM) in HBE/p53i cells **D.** Immunofluorescence staining for the detection of NNK-induced IGF-1R phosphorylation. Cells were stimulated with NNK (10 μM) for 24 h. **E.** Up-regulation of IGF-1R phosphorylation in HCC-15 cells as determined by fluorescence microscopy.

Our previous study showed that IGF-1R is activated in human high-grade dysplasia tissues and in NNK-exposed murine lung tissues [11]. Recently, we found that NNK stimulates IGF-1R activation in lung epithelial cells, promoting tumor formation (manuscript submitted for publication). We showed that NNK induced a rapid IGF-1R activation in primary cultured normal human lung epithelial (HBE) cells derived from large airways, in various immortalized, normal HBE cell lines, including BEAS-2B, and in premalignant HBE cell line carrying loss of p53 expression (HBE/p53i). In the current study, we confirmed that IGF-1R is activated in a sustained manner during chronic exposure to NNK. HBE/p53i and BEAS-2B cells displayed time-dependent increases in IGF-1R phosphorylation with a maximal phosphorylation at 24 h after stimulation with NNK (Figure 1A). NNK also induced a dose-dependent upregulation of IGF-1R phosphorylation in BEAS-2B cells (Figure 1B). The NNK treatment stimulated the IGF-1R signaling cascade as shown by the increased phosphorylation of protein kinase B (Akt) (Figure 1C). Immunofluorescence staining further revealed increases in IGF-1R phosphorylation in HBE/p53i and BEAS-2B cells in response to the NNK treatment for 24 h (Figure 1D). Because IGF-1R and IR are structurally similar to each other (84% homology in the cytoplasmic β subunit, which contains intrinsic tyrosine kinase activity) [12], commercially available antibodies detecting phosphorylated IGF-1R cross-react with phosphorylated IR. Hence, we confirmed that NNK induced IGF-1R phosphorylation by utilizing HCC-15 non-small cell lung cancer cells that lack IR expression (Figure 1E).

### Association of β-adrenergic receptor with NNK-induced IGF-1R phosphorylation in lung epithelial cells

**Figure 2:**
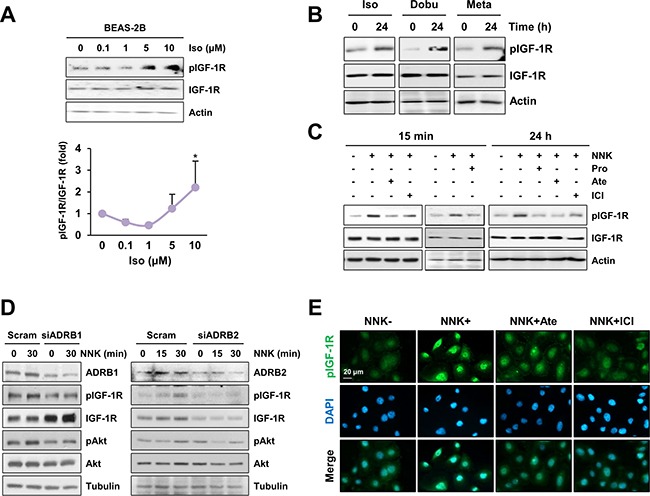
β-AR is involved in NNK-mediated IGF-1R phosphorylation **A.** Immunoblot analysis demonstrating a dose-dependent increase in IGF-1R phosphorylation by treatment with NNK in BEAS-2B cells (*n* = 3 per group). Densitometric analysis of total and phosphorylated IGF-1R blots (normalized to actin) was performed using the Image J software. Data are presented as the mean ± SD. The statistical significance of difference was determined by Student's *t*-test (*: *P* < 0.05; ***: *P* < 0.001). Iso: isoproterenol. **B.** Immunoblot analysis demonstrating the activation of IGF-1R by stimulation with β-AR agonists (10 μM) in BEAS-2B cells. Iso: isoproterenol; Dobu: dobutamine; Meta: metaproterenol. **C.** Inhibition of NNK-induced IGF-1R phosphorylation by treatment with β-AR antagonists (10 μM) in BEAS-2B cells was analyzed by Western blot analysis. Pro: propranolol; Ate: atenolol; ICI: ICI-118,551. Cells were stimulated with NNK for 15 min (left) or 24 h (right) in the presence or absence of indicated inhibitors. In case of a short-term NNK stimulation, cells were pretreated with inhibitors for 3 h. **D.** Immunoblot analysis evaluating the suppression of IGF-1R phosphorylation by silencing β1- or β2-AR expression in HBE/p53i cells. **E.** Immunofluorescence staining to detect the modulation of IGF-1R phosphorylation by blockade of β-AR in HBE/p53i cells. Cells were treated with NNK (10 μM) in the absence or presence of atenolol (Ate; 10 μM) or ICI-118,551 (ICI; 10 μM) for 15 min.

We investigated the mechanism underlying NNK-induced IGF-1R phosphorylation. Because NNK has been shown to bind β-adrenergic receptor (β-AR) [13], we investigated the possible involvement of β-AR in NNK-induced IGF-1R phosphorylation. We first assessed whether β-AR agonists, including isoproterenol (nonselective), dobutamine (selective to β1-AR), and metaproterenol (selective to β2-AR), induces IGF-1R activation in BEAS-2B cells. Isoproterenol induced a dose-dependent increase in IGF-1R phosphorylation, and activation of IGF-1R was evident with isoproterenol doses as low as 5 μM (Figure 2A). In addition, treatment with isoproterenol, dobutamine, and metaproterenol for 24 h displayed a substantial IGF-1R phosphorylation in BEAS-2B cells (Figure 2B).

To assess the involvement of β-AR in NNK-induced IGF-1R phosphorylation, we tested the effects of the blockade of β-AR on NNK-induced IGF-1R phosphorylation. Treatment with propranolol (a nonselective β-AR antagonist), atenolol (a β1-AR antagonist) or ICI-118,551 (a β2-AR antagonist) effectively suppressed IGF-1R phosphorylation during NNK exposure in BEAS-2B cells (Figure 2C). Moreover, HBE/p53i cells transfected with either β1-AR or β2-AR siRNAs exhibited markedly decreased phosphorylation of IGF-1R and Akt upon NNK exposure compared with those transfected with control scrambled siRNA (Figure 2D). Immunofluorescence staining also confirmed that NNK induced a substantial increase in IGF-1R phosphorylation, which was attenuated by pretreatment with ICI-118,551 or atenolol (Figure 2E). Together, these results indicate that β-AR is associated with NNK-induced IGF-1R phosphorylation.

**Figure 3:**
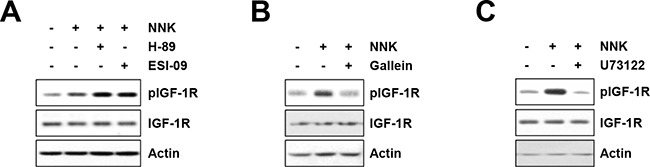
The Gβγ-mediated PLC activation mediates NNK-induced IGF-1R phosphorylation **A–C.** Suppression of NNK (10 μM)-induced IGF-1R phosphorylation by blocking Gβγ subunit and PLC but not PKA and Epac in HBE/p53i cells, as determined by Western blot analysis. HBE/p53i cells were pretreated with H-89 (A; 10 μM), ESI-09 (A; 10 μM), gallein (B; 10 μM), and U73122 (C; 1 μM) for 3 h, and then were stimulated with NNK for 15 min. The level of total and phosphorylated IGF-1R was evaluated by Western blot analysis.

We next investigated the role of downstream signaling pathways of β-AR in NNK-induced IGF-1R phosphorylation. β-AR is a G protein-coupled receptor (GPCR) that consists of Gα_s_, Gβ, and Gγ subunits [14]. Activated β-AR results in the dissociation of Gβγ subunits from the Gα subunit; the Gα_s_ subunit activates adenylyl cyclase and converts ATP to cyclic AMP (cAMP), mediating the activation of downstream effectors, including protein kinase A (PKA) and Epac [14]. The Gβγ subunits modulate the function of several effectors, including phospholipase C (PLC; PLCβ and PLCε), PI3Kγ, and calcium channels, and regulating calcium mobilization and signal transduction to promote cell proliferation and survival [15]. Accordingly, the effectors responsible for NNK-induced IGF-1R phosphorylation were determined using pharmacological inhibitors – i.e., H-89 (a PKA inhibitor), ESI-09 (an Epac inhibitor), U73122 (a PLC inhibitor), and gallein (a Gβγ subunit inhibitor). Treatment of HBE/p53i cells with H-89 or ESI-09 displayed no obvious effect on NNK-mediated IGF-1R phosphorylation (Figure 3A), whereas treatment with gallein (Figure 3B) or U73122 (Figure 3C) effectively decreased NNK-induced IGF-1R phosphorylation. Collectively, these results suggest that NNK-induced activation of the β-AR signaling pathway followed by Gβγ-mediated PLC activation is crucial for IGF-1R phosphorylation.

### β-AR activation by NNK induced *IGF2* transcription through STAT3 and NF-κB activation

**Figure 4:**
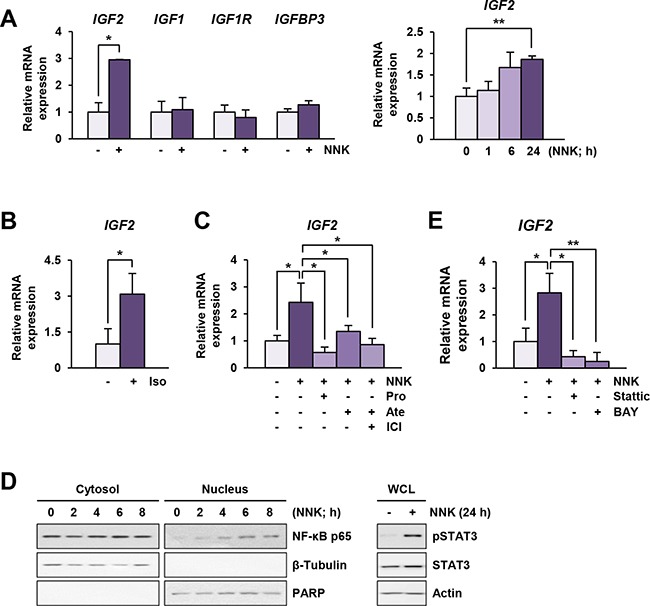
NNK induces an increase in *IGF2* transcription via β-AR, STAT3 and NF-κB activation **A** and **B.** Real-time PCR analyses demonstrating *IGF2*, but not *IGF1*, *IGF1R*, and *IGFBP3*, transcription by stimulation with NNK (10 μM) (A) or isoproterenol (Iso; 10 μM) (B) (*n* = 3 per group) in BEAS-2B cells. Data are presented as the mean ± SD. Statistical significance of difference was determined by Student's *t*-test or one-way ANOVA (*: *P* < 0.05; **: *P* < 0.01). **C.** Suppression of NNK-induced *IGF2* transcription by treatment with β-AR antagonists in BEAS-2B cells, as analyzed by real-time PCR (*n* = 3 per group). Data are presented as the mean ± SD. Statistical significance was determined by one-way ANOVA (*: *P* < 0.05). Cells were stimulated with NNK (10 μM) in the absence or presence of β-AR antagonists (10 μM) for 24 h. Pro: propranolol; Ate: atenolol; ICI: ICI-118,551. **D.** NNK-mediated phosphorylation of STAT3 and nuclear translocation of a NF-κB p65 subunit in BEAS-2B cells were analyzed by Western blot analysis. **E.** Suppression of NNK-induced *IGF2* transcription by treatment with Stattic (2 μM) or BAY 11-7082 (BAY; 5 μM) in BEAS-2B cells was analyzed by real-time PCR (*n* = 3 per group). Cells were stimulated with NNK (10 μM) with or without inhibitors for 24 h. Data are presented as the mean ± SD. Statistical significance was determined by one-way ANOVA (*: *P* < 0.05; **: *P* < 0.01).

We investigated the mechanisms underlying NNK-induced IGF-1R phosphorylation *via* the β-AR signaling pathway. We found that exposure to NNK stimulated the regulatory pathway of IGF2 secretion within 5 min, leading to a rapid activation of IGF-1R in lung epithelial cells (manuscript submitted for publication). In the current study, we further assessed whether sustained exposure to NNK modulated the mRNA expression levels of *IGF2*. The transcription of IGF-1R signaling components, including *IGF1*, *IGF1R*, and *IGFBP3*, remained unchanged in BEAS-2B cells after treatment with NNK for 24 h (Figure 4A, left panel). In contrast, BEAS-2B cells treated with NNK exhibited a significant up-regulation of *IGF2* transcription (Figure 4A, left panel). A substantial increase in *IGF2* transcription was observed as early as 6 h after NNK treatment, and the increase was statistically significant after 24 h of NNK exposure (Figure 4A, right panel). Stimulation with the β-AR agonist isoproterenol also increased *IGF2* transcription (Figure 4B), and treatment with β-AR antagonists significantly suppressed NNK-induced increases in *IGF2* mRNA expression (Figure 4C). These findings indicate that transcriptional up-regulation of *IGF2* via β-AR is responsible at least in part for the NNK-induced IGF-1R phosphorylation in lung epithelial cells.

We investigated the mechanism underlying the NNK-mediated increase in *IGF2* mRNA expression. Based on our recent findings and previous reports showing 1) direct binding of STAT3 to *IGF2* P3 and P4 promoters [16], 2) NF-κB-mediated up-regulation of *IGF2* transcription [17], and 3) activation of STAT3 and NF-κB by NNK [18], we examined the involvement of STAT3 and NF-κB in NNK-induced *IGF2* transcription. Indeed, NNK increased the nuclear translocation of NF-κB p65 subunit and the level of phosphorylated STAT3 (pSTAT3) expression (Figure 4D). Moreover, treatment with the STAT3 inhibitor Stattic or the NF-κB inhibitor BAY 11-7082 significantly down-regulated NNK-stimulated *IGF2* transcription (Figure 4E). These results suggest that activation of STAT3 and NF-κB is associated with NNK-induced *IGF2* transcription.

### Suppression of transformed phenotypes and in vivo tumor formation by blockade of β-AR

**Figure 5:**
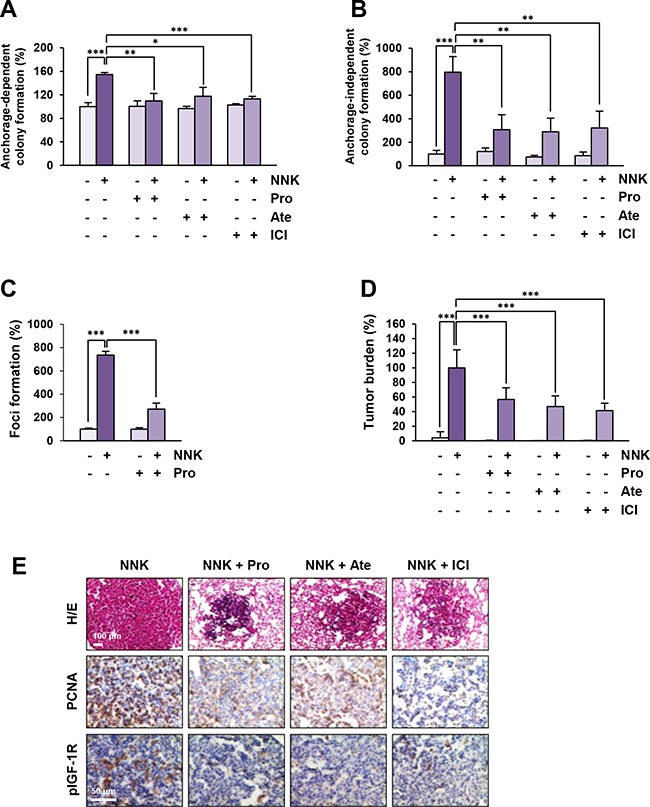
Blockade of β-AR suppresses NNK-induced cell transformation and murine lung tumor formation **A** and **B.** Decreases in anchorage-dependent (A; *n* = 3 per group) and –independent (B; *n* = 4 per group) colony formation of BEAS-2B cells by treatment with β-AR antagonists. Data are presented as mean ± SD. Statistical significance was determined by one-way ANOVA (*: *P* < 0.05; **: *P* < 0.01; ***: *P* < 0.001). **C.** Inhibition of NNK-induced foci formation of HBE/p53i cells by treatment with propranolol (*n* = 3 per group). Data are presented as mean ± SD. Statistical significance was determined by one-way ANOVA (***: *P* < 0.001). **D.** Suppression of the NNK-mediated increase in tumor burden by blockade of β-AR (*n* = 7-14 per group). Data are presented as mean ± SD. Statistical significance was determined by one-way ANOVA (***: *P* < 0.001). **E.** Immunohistochemical analysis demonstrating the suppression of IGF-1R phosphorylation and cell proliferation (determined by PCNA expression) in a nodule of the lung from mice treated with NNK and β-AR antagonists. Pro: propranolol; Ate: atenolol; ICI: ICI-118,551.

To investigate whether blockade of β-AR could inhibit the NNK-induced acquisition of transformed phenotypes in lung epithelial cells, we examined the effects of β-AR antagonists on NNK-mediated transformed phenotypes, including anchorage-dependent and -independent colony formation and foci formation resulting from single-cell survival, anchorage-independent growth, and loss of contact inhibition, respectively [19–21]. HBE cells exposed to NNK formed significantly more colonies than those of vehicle-treated control cells, and treatment with β-AR antagonists substantially suppressed the NNK-induced anchorage-dependent (Figure 5A) and anchorage-independent (Figure 5B) colony-forming abilities. The NNK-treated HBE cells also exhibited a significantly increased ability to form foci, and blockade of β-AR markedly suppressed the NNK-induced foci formation (Figure 5C). These results suggest that β-AR inhibition suppresses NNK-mediated HBE cell transformation.

Finally, we investigated whether blocking β-AR could inhibit NNK-induced lung tumor formation in vivo. Female A/J mice were treated by oral gavage with vehicle (PBS) or NNK, either alone or together with β-AR antagonists for 20 weeks. As shown in Figure 5D, mice treated with NNK alone displayed a profoundly increased tumor burden compared with the control group, and the tumor burden was significantly decreased by the administration of β-AR antagonists. As shown by the results from immunohistochemical analysis, IGF-1R phosphorylation and PCNA expression, a marker of cell proliferation [22], were markedly lower in the lungs of mice treated with β-AR antagonists than those from control mice (Figure 5E). Therefore, along with the in vitro results, these in vivo results suggest that blockade of β-AR effectively suppresses NNK-mediated lung tumor formation by inhibiting IGF2-mediated IGF-1R phosphorylation.

## DISCUSSION

In this study, we demonstrated that 1) β-AR mediates NNK-induced IGF-1R activation through the up-regulation of *IGF2* transcription; 2) the Gβγ subunits dissociated from β-AR upon NNK exposure stimulate IGF-1R through the PLC-mediated pathway; and 3) the inhibition of β-AR suppresses NNK-mediated malignant cell transformation and tumor formation in mice. These results suggest that inhibition of NNK-induced IGF2-IGF-1R activation by blocking β-AR could be a novel approach for the chemoprevention of smoking-associated lung cancer.

Despite extensive investigation to develop effective strategies for the prevention of lung cancer, smoking cessation is the only way to prevent the onset or progression of lung cancer [7]. The alteration of several genes is implicated in the carcinogenic process of lung cancer [7]; therefore, targeting these mutated genes or related signaling pathways is an efficacious approach for lung cancer prevention. Although *EGFR* and *KRAS* are frequently mutated in non-smoking- and smoking-related lung cancer, respectively, and thus can be considered targets for lung cancer prevention, their direct modulation by chemicals is not applicable for lung cancer prevention due to potential side effects and toxicity when applied as long-term treatment. Hence, considering that smoking remains the primary cause of lung cancer, targeting signal transduction that can be modulated by a smoking component is a plausible approach for lung cancer prevention.

The activation of receptor tyrosine kinases (RTKs) is strongly implicated in oncogenesis, cancer progression, and drug resistance [23]. Of these processes, IGF-1R signaling is critical for cell proliferation, survival, invasion, and angiogenesis [24]. Our previous study demonstrated the association of IGF-1R activation with lung carcinogenesis [11]. We found that IGF-1R signaling was activated in premalignant and malignant human lung tissues (high-grade dysplasia and metaplasia) and NNK-exposed lung epithelial cells and murine lung tissues [11]. These results suggest that IGF-1R may be an important target for the prevention of smoking-induced lung cancer formation. However, inhibition of IGF-1R directly is not applicable for prevention because of the potential side effects of small-molecule IGF-1R inhibitors, such as hyperglycemia due to cross-reactivity to the insulin receptor (IR) [25]. Hence, suppression of signaling networks responsible for aberrant IGF-1R signaling activation by smoking would be more suitable for further clinical utility; however, the underlying mechanisms have been poorly investigated. Therefore, the present study aimed to explore alternative means to control the IGF-1R pathway by investigating the mechanisms involved in NNK-induced IGF-1R activation.

We found that the β-AR pathway is activated by exposure to NNK, leading to IGF-1R activation. It has been proposed that various RTKs, including EGFR and IGF-1R, can be transactivated by GPCRs via several mechanisms, including ligand-dependent activation by metalloproteinase-dependent shedding of ligands and reactive oxygen species (ROS)- or Src-mediated ligand-independent activation [26]. RTK transactivation via direct association between RTKs and GPCRs through the formation of a complex with other non-receptor tyrosine kinases, adaptor proteins, and β-arrestin has also been proposed [27, 28]. Our results demonstrate that NNK treatment leads to increased *IGF2* transcription via activating β-AR, STAT3, and NF-κB. Together with our recent observation on the association of the β-AR pathway with rapid IGF-1R phosphorylation via increased IGF2 secretion (manuscript submitted for publication), exposure to NNK is likely to stimulate IGF-1R phosphorylation via dual pathways through a rapid increase in IGF2 secretion followed by a transcriptional increase in *IGF2* expression.

Our results demonstrate blockade of NNK-induced IGF-1R activation via genomic or pharmacological approaches that block β-AR, Gβγ, or PLC. Considering that Gβγ-mediated PLC and subsequent IP_3_R activation play important roles in the increase in intracellular calcium [29] and STAT3 and NF-κB are known to be modulated by a calcium/calmodulin-dependent protein kinase II [30, 31], Gβγ-mediated PLC activation couple the NNK-stimulated β-AR signaling pathway with IGF2 regulation, resulting in activation of the IGF-1R signaling pathway. These results appear to be novel and distinct from the previously reported IGF-1R transactivation. Most importantly, our data provide evidence that blockade of the β-AR signaling pathway effectively suppresses NNK-stimulated lung tumor formation in mice. These findings corroborate the in vitro studies, supporting the novel concept that blocking the β-AR signaling pathway is an alternative strategy to suppress aberrant IGF-1R activation and, thus, prevent lung cancer formation in smokers. Indeed, β-blockers (β1-AR antagonists) have been widely used for the treatment of hypertension, and the evaluation of their efficacy and safety has been thoroughly tested; therefore, the use of β-blockers can be an effective approach for cancer prevention. In agreement with this notion, β-AR has also emerged as an attractive target for adjuvant cancer therapy based on its intrinsic role as a receptor of stress-associated neurotransmitters – i.e., epinephrine and norepinephrine – and its downstream signaling pathways responsible for cell proliferation, survival, metastasis, and angiogenesis [32]. In addition, the inhibition of β-AR provides better therapeutic responses to conventional anticancer therapies in neuroblastoma, hemangioendothelioma, and angiosarcoma [33, 34]. Moreover, although controversial results have also been reported [35], the use of β-blockers may reduce the mortality of lung, breast, ovary, and prostate cancer [36–40]. Although the correlation between the use of β-blockers and the reduction of cancer risk remains questionable and needs to be further investigated [41, 42], our results provide preclinical evidence for using β-AR antagonists in prevention of smoking-associated lung cancer. In addition, previous studies have reported a possible positive correlation between hypertension and lung cancer in current smokers [43, 44]. Considering the traditional use of β-blockers as a therapy for hypertension, the relationship between smoking-related lung cancer and hypertension also needs to be examined.

In summary, the present study demonstrates that NNK activates IGF-1R through the β-AR-mediated activation of Gβγ-PLC and up-regulation of *IGF2* transcription via activation of β-AR, STAT3, and NF-κB. Blockade of β-AR significantly reduced the acquisition of transformed phenotypes in lung epithelial cells and NNK-induced lung tumor formation in mice. These results suggest that blockade of β-AR may be efficacious for the prevention of smoking-related lung cancer. Further studies are warranted to investigate the effectiveness of blockade of β-AR in smoking-associated lung cancer chemoprevention using additional preclinical and clinical settings.

## MATERIALS AND METHODS

### Cell culture

Human bronchial epithelial (HBE) cells knocked down for p53 expression using RNA interference (HBE/p53i) [45] were kindly provided by Dr. John D. Minna (The University of Texas Southwestern Medical Center, Dallas, TX, USA). BEAS-2B cells were purchased from American Type Culture Collection (ATCC; Manassas, VA, USA). These cells were cultured in K-SFM (Invitrogen, Grand Island, NY, USA) supplemented with 5 ng/ml recombinant epidermal growth factor (EGF) and 50 μg/ml bovine pituitary extracts. A549 and HCC-15 cells, purchased from ATCC, were cultured in RPMI 1640 supplemented with 10% fetal bovine serum and antibiotics (all from Welgene, Daegu, Republic of Korea). Cells were maintained at 37°C with 5% CO_2_ in a humidified atmosphere.

### Reagents

Dimethyl sulfoxide (DMSO), crystal violet, 3-(4,5-dimethyltrizaol-2-yl)-2,5-diphenyltetrazolium bromide (MTT), and other chemicals, unless otherwise indicated, were purchased from Sigma-Aldrich (St. Louis, MO, USA). 4-(Methylnitrosamino)-1-(3-pyridyl)-1-butanone (NNK) was purchased from Sigma or Toronto Research Chemicals (TRC; Toronto, ON, Canada). Isoproterenol, dobutamine, metaproterenol, propranolol, atenolol, ICI-118,551, gallein, and U73122 were purchased from Tocris Bioscience (Bristol, UK). ESI-09 was purchased from Enzo Life Sciences (Farmingdale, NY, USA). Antibodies against phosphorylated IGF-1R (pIGF-1R) at Y1131 or Y1135/36, IGF-1R, phosphorylated STAT3 at Y705, STAT3, phosphorylated Akt at S473, Akt, β-tubulin, α/β-tubulin (tubulin), and PARP were purchased from Cell Signaling Technology (Danvers, MA, USA). Antibodies against actin, β1-AR, and β2-AR were purchased from Santa Cruz Biotechnology (Santa Cruz, CA, USA). Anti-proliferating cell nuclear antigen (PCNA) antibody was purchased from Abcam (Cambridge, MA, USA).

### Anchorage-dependent colony formation assay

BEAS-2B cells were seeded into 6-well plates at a density of 300 cells/well and were incubated for 24 h. Cells were stimulated with 10 μM NNK in the presence or absence of inhibitors for 10 days. Colonies were stained with 0.01% crystal violet and were counted.

### Anchorage-independent colony formation assay

Before seeding, the base agar (final agar concentration: 1%) was prepared in 24-well plates. BEAS-2B cells (final 2 x 10^3^ cells/well) were mixed with sterile low-melting agar solution and immediately poured onto the base agar. NNK (10 μM) with or without inhibitors diluted in 0.5 ml of complete medium were added. Cells were incubated for 3 weeks, and the drug-containing medium was replaced every 3-4 days. Colonies were stained with the MTT solution (final 500 μg/ml), photographed, and counted.

### Foci formation assay

HBE/p53i cells at 70-80% confluence in 6-well plates were treated with NNK (10 μM) with or without inhibitors for 2 weeks. The drug-containing media were replaced every 2-3 days. After incubation, foci were photographed and counted.

### Preparation of cytosol/nuclear extracts

BEAS-2B cells were stimulated with NNK for the indicated time points. Cytosol fraction was prepared with hypotonic Buffer A [10 mM HEPES (pH 7.9), 1.5 mM MgCl_2_, 10 mM KCl, 0.5 mM DTT, 1 mM EDTA, protease inhibitor cocktail (Roche Applied Sciences, Indianapolis, IN, USA) and phosphatase inhibitor cocktail (Roche)]. After washing with Buffer A, pellets were suspended in hypertonic Buffer C [20 mM HEPES (pH 7.9), 1.5 mM MgCl_2_, 420 mM NaCl, 0.5 mM DTT, 0.2 mM EDTA, 25% glycerol, protease inhibitor cocktail, and phosphatase inhibitor cocktail]. After centrifugation, supernatants were collected and stored at −70°C. The cytosolic and nuclear localization of the NF-κB p65 subunit was determined by Western blot analysis as described below.

### Western blot analysis

Cells were seeded into 6-well plates at a density of 3 × 10^5^ cells/well. On the next day, culture media were replaced by supplement-free K-SFM (for HBE/p53i and BEAS-2B), and the cells were incubated for 1-2 days. When necessary, the cells were pretreated with inhibitors 3-6 h before stimulation and then stimulated with NNK (10 μM) or β-AR agonists (10 μM) for the indicated times. Cells were harvested with modified RIPA lysis buffer [50 mM Tris-HCl (pH 7.4), 150 mM NaCl, 1 mM EDTA, 0.25% sodium deoxycholate, 1% Triton X-100, 100 mM NaF, 1 mM Na_3_VO_4_, 1 mM phenylmethylsulfonyl fluoride (PMSF), 1 μg/ml aprotinin, and 1 μg/ml leupeptin]. Equal amounts of proteins were resolved by 8% SDS-PAGE and then electrically transferred onto a PVDF membrane (ATTO Corp., Osaka, Japan). Membranes were blocked with blocking buffer [3% BSA in Tris-buffered saline (TBS) containing 0.1% Tween-20 (TBST)] for 1 h at room temperature, and then were incubated with primary antibodies diluted in blocking buffer (1:1,000) overnight at 4°C. After washing several times with TBST, membranes were incubated with corresponding secondary antibodies diluted in 3% non-fat dry milk in TBST (1:5,000) for 1-2 h at room temperature. Membranes were washed several times with TBST and were visualized using an enhanced chemiluminescence (ECL) detection kit (Thermo Fisher Scientific, Grand Island, NY, USA). When necessary, densitometric analysis was performed using the Image J software (National Institutes of Health, Bethesda, MD, USA).

### Transfection

For transient knockdown of β1- or β2-ARs, cells were transfected with scrambled or gene-specific targeting small interfering RNAs (siRNAs; purchased from Integrated DNA Technologies, Coralville, Iowa, USA) using Lipofectamine RNAiMAX (Invitrogen, Carlsbad, CA, USA) according to the manufacturer's instructions. Knockdown of these genes was confirmed by Western blot analysis.

### Reverse transcription-polymerase chain reaction (RT-PCR)

RT-PCR and real-time PCR analyses were performed as described previously [46]. The primer sequences used for this study are described in Table 1.

**Table 1:** Primer sequences used for real-time PCR analysis

Gene	Forward (5′-3′)	Reverse (5′-3′)
*IGF1*	ATGTATTGCG CACCCCTCAA	GGGCACGG ACAGAGCG
*IGF2*	CCGTGCTTCC GGACAACTT	CTGCTTCCAG GTGTCATATTGC
*IGF1R*	TGAAAGTGACG TCCTGCATTTC	GGTACCGGTG CCAGGTTATG
*IGFBP3*	TCTGCGTCAA CGCTAGTGC	GCTCTGAGACT CGTAGTCAACT
*ACTB*	GCGAGAAGAT GACCCAGATC	GGATAGCAC AGCCTGGATAG

### Animal experiments

All of the animal experiments were performed using protocols approved by the Seoul National University Institutional Animal Care and Use Committee. Five-week-old A/J mice were randomly grouped and exposed to NNK (3 μmol; dissolved in sterile PBS) twice per week. After 2-4 weeks, several inhibitors (propranolol: 1 mg/kg; atenolol: 5 mg/kg; ICI-118,551: 0.5 mg/kg; drugs were dissolved in sterile PBS) were administered with or without NNK by oral gavage for an additional 20 weeks. The mice were euthanized, and tumor formation was evaluated and compared with that of the vehicle-treated control group. After gross evaluation of tumor formation, microscopic evaluation of formalin-fixed and paraffin-embedded lung tissue stained with hematoxylin and eosin (H&E) was performed to measure the mean tumor number (N) and volume (V) in a blinded fashion. The tumor volume was calculated using the following formula: V (mm^3^) = (long diameter × short diameter^2^)/2. The tumor burden was calculated using the following formula: mean tumor number (N) × mean tumor volume (V). The number and size of tumors were calculated in five sections uniformly distributed throughout each lung.

### Immunofluorescence and immunohistochemistry

Immunofluorescence and immunohistochemical analyses to detect phosphorylated IGF-1R or PCNA expressions were performed as described previously [46].

### Statistics

The data are presented as the mean ± SD. All in vitro experiments were independently performed at least twice, and a representative result is presented. The statistical significance was analyzed using a two-sided Student's *t*-test or one-way analysis of variance (ANOVA) followed by Dunnett's *post-hoc* test using GraphPad Prism 6 (GraphPad Software Inc., La Jolla, CA, USA). *P* values less than 0.05 were considered statistically significant.
